# The feasibility of matching on a propensity score for acupuncture in a prospective cohort study of patients with chronic pain

**DOI:** 10.1186/s12874-017-0318-4

**Published:** 2017-03-16

**Authors:** Eric S. Johnson, John F. Dickerson, William M. Vollmer, Alee M. Rowley, Cheryl Ritenbaugh, Richard A. Deyo, Lynn DeBar

**Affiliations:** 1 The Center for Health Research, Kaiser Permanente Northwest, 3800 North Interstate Avenue, Portland, OR 97227-1099 USA; 2 Department of Family and Community Medicine, The University of Arizona, 1450 North Cherry Avenue, Tucson, AZ 85719 USA; 3 Department of Family Medicine, Oregon Health and Science University, Mail Code FM, 3181 Sam Jackson Road, Portland, OR 97239 USA

**Keywords:** Propensity score, Prospective cohort, Pain, Acupuncture, Comparative effectiveness

## Abstract

**Background:**

Propensity scores are typically applied in retrospective cohort studies. We describe the feasibility of matching on a propensity score derived from a retrospective cohort and subsequently applied in a prospective cohort study of patients with chronic musculoskeletal pain before the start of acupuncture or usual care treatment and enrollment in a comparative effectiveness study that required patient reported pain outcomes.

**Methods:**

We assembled a retrospective cohort study using data from 2010 to develop a propensity score for acupuncture versus usual care based on electronic healthcare record and administrative data (e.g., pharmacy) from an integrated health plan, Kaiser Permanente Northwest. The propensity score’s probabilities allowed us to match acupuncture-referred and non-referred patients prospectively in 2013-14 after a routine outpatient visit for pain. Among the matched patients, we collected patient-reported pain before treatment and during follow-up to assess the comparative effectiveness of acupuncture. We assessed balance in patient characteristics with the post-matching c-statistic and standardized differences.

**Results:**

Based on the propensity score and other characteristics (e.g., patient-reported pain), we were able to match all 173 acupuncture-referred patients to 350 non-referred (usual care) patients. We observed a residual imbalance (based on the standardized differences) for some characteristics that contributed to the score; for example, age, -0.283, and the Charlson comorbidity score, -0.264, had the largest standardized differences. The overall balance of the propensity score appeared more favorable according to the post-matching c-statistic, 0.503.

**Conclusion:**

The propensity score matching was feasible statistically and logistically and allowed approximate balance on patient characteristics, some of which will require adjustment in the comparative effectiveness regression model. By transporting propensity scores to new patients, healthcare systems with electronic health records can conduct comparative effectiveness cohort studies that require prospective data collection, such as patient-reported outcomes, while approximately balancing numerous patient characteristics that might confound the benefit of an intervention. The approach offers a new study design option.

## Background

Propensity scores are an increasingly popular method of controlling potential confounding in observational studies that compare the effectiveness of healthcare interventions [1, 2]. Propensity scores are typically used in retrospective cohort studies and involve fitting regression models to predict treatment groups based on selected characteristics derived from administrative healthcare data or electronic health record (EHR) data [2].

To date, propensity score methods have not been used to recruit and match subjects on an ongoing basis in prospective cohort studies that require the collection of patient-reported outcomes (PROs). PROs may be required at baseline (e.g., to define cohort eligibility or assess treatment effect heterogeneity) or may be required as an outcome. Examples of such PRO data include self-reported measures of depression or pain. While the systematic collection of PROs in the EHR remains uncommon in routine practice settings, collection of PROs is increasing [3].

This paper details a novel use of a propensity score to permit ongoing matching in a prospective cohort study comparing the effectiveness of acupuncture versus usual care to treat pain in patients with chronic musculoskeletal pain (CMP). A propensity score offered a more efficient approach statistically and logistically for enrollment in the prospective cohort study than alternative study designs. We sought to match on patients’ pre-treatment Brief Pain Inventory score because it served as an outcome for evaluating the comparative effectiveness of acupuncture. The Brief Pain Inventory could only be collected by interviewing patients; it was not collected systematically in routine practice. Had we matched on Brief Pain Inventory score and enrolled patients in the cohort for additional prospective data collection of patient reported outcomes, we could have developed a propensity score after enrollment finished to adjust for additional patient characteristics that might confound the acupuncture effect. Some patients on whom we would have collected patient-reported outcomes would have been excluded from the analysis because of a lack of overlap in their propensity scores (i.e., trimming), which would have resulted in a loss statistically (worse precision) and a loss logistically (wasted research staff effort). Given a pre-determined level of research funding, we sought to invest in prospective data collection for those patients who would contribute to the estimate of comparative effectiveness. Using recent historical EHR and other administrative healthcare data, we developed a propensity score to predict the probability of referral and initiation of acupuncture in patients with CMP. We matched patients by deciles of propensity as we enrolled them in the prospective cohort study to reduce imbalance in the final comparison groups. Our paper describes the feasibility of our approach and assesses the degree of balance in baseline characteristics that we achieved.

## Methods

Our prospective cohort study, the RELIEF study, has been described elsewhere [4]. Although the design of RELIEF involved parallel studies of both acupuncture and chiropractic care for the management of CMP, this report only describes the acupuncture cohort study. The prospective cohort study included one cohort of patients who were referred for acupuncture and a second cohort of patients who were not referred for acupuncture, which served as the control cohort. All study procedures and the study protocol were approved by the Kaiser Permanente Northwest (KPNW) Institutional Review Board (IRB). KPNW is an integrated delivery system that managed healthcare for patients in Oregon and Washington States in the northwestern United States. KPNW served as the study’s setting. For the prospective cohort study—the only portion of the study reported here that required direct patient contact and consenting—the KPNW IRB granted a waiver of signed informed consent and an alteration of the privacy rule authorization (Health Insurance Portability and Accountability Act, HIPAA; no signature). Participants provided verbal consent and HIPAA elements were reviewed verbally with all participants who enrolled in the prospective cohort study. For the retrospective cohort studies, KPNW’s IRB granted a waiver of signed informed consent and an alteration of the privacy rule authorization. The National Center for Complementary and Alternative Medicine at the National Institutes of Health sponsored the study. The sponsor had no role in the study design, data collection, data analysis, data interpretation, writing of the report or decision to submit for publication.

### Use of retrospective cohorts to develop and validate the propensity score

We identified adult members at KPNW, an integrated delivery system with approximately 480,000 members, who had a diagnosis for chronic pain documented in the EHR on at least three pain-related outpatient visits over a six to 18-month period. The pain had to be chronic in that it occurred over at least six months. We allowed up to 18 months for patients to meet the criteria for three pain-related visits. One of the three pain-related visits had to include a diagnosis of chronic musculoskeletal pain. We excluded patients with cancer, dementia, psychoses (or transient psychotic episodes), and those in hospice.

From that source population, we identified patients with subsequent referral and documented use of acupuncture during 2010. We also identified a comparison group of patients who met the same eligibility criteria but who had no evidence of subsequent referral and use of acupuncture after their most recent visit for chronic pain in 2010. We then excluded patients with a recent prior referral for acupuncture or use of acupuncture (within the past six months) because a valid propensity score requires incident users [5]. Patients were insured by KPNW continuously for at least six months before the index primary care visit for chronic pain (i.e., the visit associated with acupuncture referral or the most recent pain-related visit for the comparison cohort).

We then developed a propensity score model to predict initiation of acupuncture using logistic regression [6]. Potential variables for inclusion in the model included information available from the EHR and related administrative databases, such as outpatient pharmacy prescription fills. A complete list of the diagnosis, procedure and medication codes is available upon request. In broad categories, the candidate characteristics included: age; the frequency of recent outpatient utilization (e.g., all outpatient visits, physical therapy visits); behaviors and symptoms (e.g., smoking, substance abuse, sleep problem); treatments for pain (e.g., medications and procedures); and outpatient diagnoses (e.g., pain-specific as well as the Charlson comorbidity score).

In deciding on the predictor characteristics for the propensity score, we consulted physicians with expertise in pain who considered whether the characteristic would be likely to predict initiation of acupuncture, and separately, to predict pain (the primary outcome for the prospective cohort to estimate comparative effectiveness). Characteristics that meet those criteria are more effective at reducing confounding and less likely to inflate the treatment effect confidence interval [7]. We excluded predictor characteristics with a very low prevalence because the coefficients in the propensity score would be less reliable (imprecise) for future application in the prospective cohort study [8]. We specified the predictor characteristics *a priori* in consultation with physicians instead of using statistical significance or other data-dependent variable reduction strategies. Our *a priori* strategy reduced optimism in the propensity score coefficients that would have reduced its effectiveness in future populations [9]. To assess how well the propensity score from 2010 might predict in subsequent populations, we conducted a temporal validation using KPNW data from 2011. The temporal validation assessed the propensity score’s ability to discriminate between future patients who initiate acupuncture and those who do not initiate acupuncture. We used the concordance statistic (c-statistic) to measure discriminative ability, which is equivalent to the area under the receiver operating characteristic curve [9]. We assessed the agreement between the observed and predicted probabilities of referral to measure calibration [9].

### Prospective cohort that matched on the propensity score

**Fig. 1:**
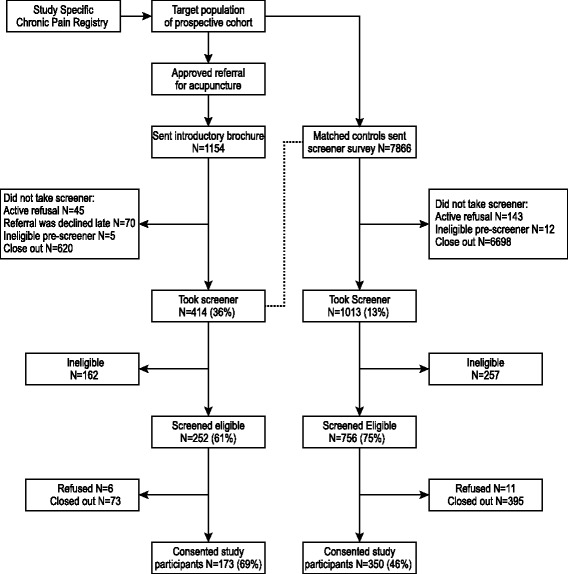
Participant flow diagram for the prospective cohort study

We were able to identify eligible patients with a referral for acupuncture during 2013 or 2014 before they began acupuncture because the EHR is updated daily. This timely identification also allowed us to recruit referred patients and to screen them for study eligibility before treatment. The eligibility criteria were similar to those defined for the retrospective cohorts described above with the following exception: we did not require patients to initiate acupuncture. For any given referral patient, we identified potential controls as chronic musculoskeletal pain patients with a recent (preceding 10 days) visit for pain that did not result in a referral for acupuncture. Staff screened all patients for eligibility by telephone or through a study website to collect patient-reported information that was not documented in the EHR. We excluded patients who reported any of the following (Fig. 1):

- Declined to be screened for the prospective cohort study
- < 4 on the (0–10 scale) for Brief Pain Inventory bothersomeness score
- Non-persistent pain
- Already started acupuncture at KPNW (i.e., we could not obtain pre-treatment measures)
- Recent undocumented use of acupuncture (i.e., outside of KPNW in past six months)
- Pregnant
- Intended to move out of state (i.e., patients would be lost to follow-up).

Among eligible patients referred for acupuncture, we calculated the probability of acupuncture initiation using the propensity score equation developed with the retrospective cohort from 2010. A patient with a referral order for acupuncture documented on Monday, for example, would be screened as soon as possible—typically within three days. If the patient were eligible according to the criteria noted above, he or she would be “scored” according to the propensity score. The predicted probability of acupuncture initiation for this patient might be 0.05, which would map to one of the deciles defined by the 2010 score.

In parallel, we recruited control patients who were not referred for acupuncture. Control patients were matched on propensity score decile as well as sex and the Brief Pain Inventory bothersomeness item (+/- two points on the 10-point scale) as assessed during screening [10]. Propensity score deciles were defined by the observed distribution rather than deciles of absolute risk (e.g., the third decile was the third decile of the propensity score distribution, and not those with predicted probability of acupuncture use between 20 and 30%). We screened up to 15 control patients per acupuncture-referred patient to obtain a match. Our goal was to match two control patients per acupuncture-referred patient. When we found more than two control patients who met our matching criteria based on the propensity score decile, sex and Brief Pain Inventory bothersomeness, we selected control patients with the most recent visit for pain. For example, if the patient referred for acupuncture had been screened for the study within three days of the referral, we sought control patients who had been screened within three days (or as close as possible) of their most recent visit for pain.

Some eligible patients who consented to the screening declined to participate in the prospective cohort study and were excluded (*n* = 79) from the analysis. The Figure illustrates the number of patients who were screened, eligible, matched, and consented to participate in the prospective study.

### Assessing balance in predictors

We calculated the standardized difference for each of the characteristics to assess the degree of the imbalance in baseline (pre-treatment) predictor characteristics that might confound estimates of comparative effectiveness. For continuous measures, the standardized difference is defined as the difference in means divided by the pooled standard deviation, and for binary data as 2*[arcsin(√P1) – arcsin(√P2)] [11]. We used the unadjusted standard deviation for the standardized differences presented in Table 1 to make the comparison between adjusted and unadjusted standardized differences more meaningful. Larger differences in the standardized differences indicate worse discrepancies.

**Table 1:** Impact of propensity score adjustment for development cohort

	Unadjusted for propensity score			Adjusted for decile of propensity score
	Started Acupuncture (*n* = 952)	Did Not Start Acupuncture (*n* = 59,564)	Standardized difference^a^	Standardized difference^a^
Propensity score characteristics^b^				
Opioid therapy plan	28.8%	17.8%	0.261	0.023
Physical therapy past 30 days	16.3%	15.1%	0.032	−0.008
Physical therapy past 31–180 days	25.0%	11.1%	0.368	0.018
Physical therapy past 181–365 days	24.5%	12.1%	0.324	0.016
Nonspecific chronic pain	29.6%	14.4%	0.373	0.031
Substance abuse	4.6%	4.1%	0.028	0.003
Sleep problem	23.6%	14.6%	0.232	0.016
History of tobacco use	14.2%	12.9%	0.036	0.000
Anxiety	23.7%	15.6%	0.206	0.017
Pain treatment procedure	38.2%	22.5%	0.344	0.020
Pain diagnosis procedure	65.3%	52.5%	0.261	0.011
Pain medication	81.2%	65.0%	0.370	0.008
Age (years)	53.8 (14.0)	55.2 (15.0)	−0.097	−0.008
Number of outpatient visits	15.9 (10.8)	10.4 (10.1)	−0.543	0.011
Months since cohort entry	29.1 (14.7)	25.2 (15.6)	−0.246	0.010
Ambulatory Charlson score	1.8 (2.2)	1.9 (2.1)	−0.036	−0.006
Demographic Characteristics				
Female	72. 8%	62.0%	0.232	0.146
White	91.2%	91.9%	−0.023	−0.033
Hispanic	5.4%	7.7%	−0.091	−0.093
Medical and Psychiatric Comorbidities				
Depression	21.5%	15.8%	0.148	0.014
Types of Nonmalignant Chronic Pain (NCP)				
Back and/or neck pain	80.6%	55.9%	0.539	0.407
Joint pain (including osteoarthritis)	79.3%	79.3%	0.001	−0.114
Fibromyalgia/other myofascial pain	30.1%	11.8%	0.461	0.257
Headaches	18.0%	12.0%	0.167	0.077
Neuropathy	1.5%	1.5%	−0.001	0.011
Temporomandibular disorders	5.0%	3.0%	0.105	0.046
Carpal tunnel syndrome	6.5%	5.8%	0.028	−0.016
Abdominal pain	6.3%	4.8%	0.068	−0.018
Other NCP	5.8%	6.6%	−0.034	−0.057
Two of above NCP types	81.1%	60.6%	0.457	0.224
Pharmacotherapy				
Any use of an opioid	19.9%	11.8%	0.223	0.025
Opioid morphine equivalent dose (MED)	0.3 (1.7)	0.2 (0.9)	0.122	−0.012
≥ 120 MED	7.9%	4.5%	0.141	−0.007
Mental health related				
Any antidepressant use	47.6%	35.4%	0.247	0.036
Any anxiolytic use	33.1%	21.2%	0.270	0.041
Any benzodiazepine use	35.4%	21.1%	0.321	0.067

^a^Standardized difference expressed as (difference in means)/(pooled standard deviation) for continuous measures and as 2*(arcsin(√P1)-arcsin(√P2)) for binary data. For propensity score decile adjusted data, standardized differences calculated using same standard deviation as for unadjusted data in order to make comparison of standardized differences with and without adjustment more meaningful

^b^Continuous data expressed as mean (standard deviation)

We also calculated c-statistics for our propensity score models. C-statistics are traditionally used to measure the discriminatory power of a predictive model, and for logistic regression are equivalent to the area under the receiver operating characteristic curve. Thus, a c-statistic of 1.0 represents perfect discrimination, while a c-statistics of 0.5 represents no discrimination between those who initiated acupuncture and those who did not initiate. The c-statistic played no role in our selection of pre-treatment characteristics (e.g., we did not prefer larger c-statistics) and should not be interpreted as a measure of how well the propensity score may control confounding [12]. Our principal interest in the c-statistic was to assess the change in the c-statistic between the propensity score development and temporal validation cohorts to understand how well the model (from 2010) transported over time to new patients (2011) [9]. For the prospective cohort study we calculated a different version of the c-statistic, the post-matching c-statistic, which assesses overall balance in patient characteristics in the propensity score [13]. For the post-matching c-statistic, values closer to 0.5 indicate better balance between the acupuncture-referred and control cohorts.

## Results

### Development of propensity score model

We used our study-specific chronic pain registry (current KPNW patients who met our EHR study definition of likely CMP) to assemble a cohort of 60,516 eligible patients in 2010. Of these, 952 (1.6%) started acupuncture during 2010. Table 1 shows the baseline characteristics of the patients who started acupuncture and those who did not start acupuncture. The characteristics are presented first for those variables included in the propensity score model, and then for selected categories of other variables.

The majority of characteristics included in the propensity score model exhibited differences between the two patient cohorts. Patients who started acupuncture, for example, were more likely to have used pain medication (81.2% vs 65.0%, with a standardized difference of 0.37). Likewise, most of the pain-related characteristics were more common among those who started acupuncture (i.e., opioid therapy plan, non-specific chronic pain, pain treatment procedure, and pain diagnosis procedure). A more general measure of comorbidity, the Charlson score (Deyo’s adaptation), showed a negligible imbalance with a standardized difference close to zero. The corresponding c-statistic for the variables in the propensity score model was 0.739, which reflects the broad imbalance between the cohorts in these variables and the propensity score model’s ability to discriminate between the cohorts. After adjusting for propensity score decile using 10 indicator variables, however, the balance of the characteristics in the propensity score model improved. All of the standardized differences were close to zero and no values were greater than 0.05 or less than -0.05.

Many of the characteristics that were not included in the propensity score model were also imbalanced between patients who started acupuncture and those who did not (Table 1). Back or neck pain and fibromyalgia (or other myofascial pain) were more common among the patients who started acupuncture. Multiple types of pain were more common among the patients who started acupuncture: 81.1% vs 60.6%, a standardized difference of 0.46. Opioid use was more common among the patients who started acupuncture: 19.9% vs 11.8%, a standardized difference of 0.223. Adjustment for propensity score decile also improved the balance for some of these characteristics.

The propensity score model developed with data from 2010 validated adequately in a distinct cohort of patients from 2011 (c-statistic = 0.708, without matching). The propensity score’s agreement between predicted and observed probabilities of acupuncture initiation was also adequate (calibration plot not shown).

### Use of the propensity score model for recruitment into the prospective cohort study

We identified 1154 patients referred for acupuncture from the study-specific chronic pain registry and invited them to be screened for the prospective cohort study (Fig. 1). Some patients agreed to be screened according to the study’s eligibility criteria; those patients took the screener’s survey. Patients were ineligible if any of the following were true:

- Patient scored < 4 on the Brief Pain Inventory’s pain bothersome scale (ranged from 0 to 10).
- Patient’s pain was not chronic.
- Patient had a recent history (past six months) of undocumented acupuncture performed outside of KPNW.
- Patient had already started acupuncture at KPNW and we could not obtain pre-treatment measures.
- Patient was pregnant.
- Patient intended to move outside of the KPNW coverage area and would be lost to follow-up.

We screened 414 (36%) patients and determined that 252 patients were eligible for the acupuncture-referred cohort. As those 252 patients became eligible we calculated their propensity scores. The matching of control patients occurred in two steps. Once we identified an eligible patient referred for acupuncture, we selected a pool of possible control patients whose propensity score probability was in the same decile as the acupuncture-referred patient. Control cohort patients were not referred for acupuncture but had a recent pain-related visit. At that stage, the possible controls were only matched on the propensity score decile and control patients were invited to be screened according to the criteria listed above. In most instances, we identified more possible controls than required to match two control patients, so the majority were closed-out and never screened (*n* = 6698). A total of 1013 control cohort patients agreed to take the screener’s survey and 257 patients were ineligible according to the criteria.

The second step in matching occurred for the 756 control cohort patients who screened eligible: Control patients had to match on sex and Brief Pain Inventory bothersome score. Some of the eligible patients were not enrolled; they were closed out of the study for the following reasons: (1) The patient could not be matched on the patient-reported Brief Pain Inventory score (which was only collected during screening) and sex; (2) the patient was no longer required as a control because we had already identified two control patients for the patient referred for acupuncture; (3) the patient experienced technical problems with the online enrollment process, which occurred more commonly in the early study recruitment. A few patients refused to participate in the prospective cohort study. After all of those considerations itemized in the Figure, 350 control cohort patients consented to the prospective cohort study. Control patients were frequency-matched to 173 acupuncture-referred patients.

Table 2 shows the baseline characteristics of the 523 patients in the prospective cohort study. The cohorts are by definition already adjusted for the propensity score because the non-referred group was matched on decile of propensity score as part of the selection process. Hence, further analytic adjustment for decile of propensity score was unnecessary. As a result, Table 2 only presents one standardized difference estimate (without additional analytic adjustment for decile of propensity score). Most of the balance achieved by traditional adjustment for propensity score in Table 1 was retained via the matching process used to select the prospective cohort study sample. For example, the overall use of any pain medication was 57.8% (acupuncture-referred) versus 56.0% (control). The standardized differences (absolute values) ranged from 0.010 (physical therapy 31 to 180 days before the start of follow-up) to 0.283 (age). After age, the least balanced characteristics ranked by standardized difference were: comorbidity (as measured by the Deyo adaptation of the Charlson score), -0.264; physical therapy in the 181 to 365 days before the start of follow-up, 0.244; an opioid therapy plan, -0.239; a sleep problem, 0.142, physical therapy in the past 30 days, -0.141, months since cohort entry, -0.114; and, a pain diagnosis procedure, -0.104. The overall balance was more reassuring with a post-matching c-statistic of 0.503 (where 0.500 would indicate perfect balance; for example, randomization assignment in a trial would not discriminate patients’ treatment). The post-matching c-statistic should be interpreted differently from the (unmatched) c-statistics reported above for the development and validation cohorts.

**Table 2:** Impact of propensity score matching for prospective cohort

	Referred for Acupuncture (*n* = 173)	Not Referred for Acupuncture (*n* = 350)	Standardized difference^a^
Propensity score characteristics^b^			
Opioid therapy plan	20.2%	30.6%	−0.239
Physical therapy past 30 days	1.2%	3.1%	−0.141
Physical therapy past 31–180 days	11.6%	8.6%	0.010
Physical therapy past 181–365 days	16.2%	8.3%	0.244
Nonspecific chronic pain	30.1%	30.0%	0.013
Substance abuse	5.2%	3.7%	0.072
Sleep problem	26.0%	20.0%	0.143
History of tobacco use	24.9%	27.1%	−0.052
Anxiety	21.7%	18.5%	−0.080
Pain treatment procedure	18.5%	22.0%	−0.087
Pain diagnosis procedure	46.2%	51.4%	−0.104
Pain medication	57.8%	56.0%	0.036
Age (years)	49.6 (11.8)	52.8 (11.5)	−0.283
Number of outpatient visits	10.3 (8.0)	10.8 (8.2)	−0.062
Months since cohort entry	42.4 (27.7)	45.5 (26.9)	−0.114
Ambulatory Charlson score	1.1 (1.4)	1.5 (2.0)	−0.264
Characteristics that did not contribute to the propensity score			
Demographic Characteristics			
Female	71.1%	73.7%	−0.059
White	88.5%	93.5%	−0.176
Hispanic	3.8%	4.1%	−0.016
Medical and Psychiatric Comorbidities			
Depression	12.7%	16.6%	−0.109
Types of Nonmalignant Chronic Pain (NCP)			
Back and/or neck pain	71.1%	60.6%	0.223
Joint pain (including osteoarthritis)	66.5%	70.9%	−0.095
Fibromyalgia/other myofascial pain	30.6%	16.9%	0.327
Headaches	20.2%	11.1%	0.252
Neuropathy	3.5%	9.1%	−0.240
Temporomandibular disorders	2.9%	2.6%	0.020
Carpal tunnel syndrome	2.9%	5.1%	−0.116
Abdominal pain	11.0%	9.4%	0.051
Other NCP	5.2%	5.1%	0.003
Two of above NCP types	74.0%	68.0%	0.132
Pharmacotherapy			
Any use of an opioid	20.2%	21.7%	−0.036
Opioid morphine equivalent dose (MED)	0.3 (1.1)	0.3 (1.1)	−0.026
≥ 120 MED	7.5%	8.6%	−0.039
Mental health related			
Any antidepressant use	54.9%	49.4%	0.110
Any anxiolytic use	28.3%	28.3%	0.001
Any benzodiazepine use	28.9%	27.1%	0.039

^a^Standardized difference expressed as (difference in means)/(pooled standard deviation) for continuous measures and as 2*(arcsin(√P1)-arcsin(√P2)) for binary data. For propensity score decile adjusted data, standardized differences calculated using same standard deviation as for unadjusted data in order to make comparison of standardized differences with and without adjustment more meaningful

^b^Continuous data expressed as mean (standard deviation)

We also evaluated characteristics that may predict pain and confound the estimate of comparative effectiveness, but were not in the propensity score. The least balanced characteristics ranked by standardized difference were: fibromyalgia or other myofascial pain, 0.327; headaches, 0.252; neuropathy, -0.240; back or neck pain, 0.223; white race, -0.176; two types of non-chronic pain, 0.132; carpal tunnel syndrome, -0.116; antidepressant medication use, 0.110, and depression, -0.109.

## Discussion

We implemented a novel application of a propensity score in which we initially developed a propensity score in one cohort of patients and then used that propensity score to match patients on an ongoing basis in a distinct prospective cohort study to evaluate the comparative effectiveness of acupuncture. Our development cohort demonstrated that providers refer—and patients initiate—acupuncture preferentially according to measured patient characteristics, including markers of chronic pain severity (e.g., pain medications generally and opioid use specifically). Our prospective cohort demonstrated that propensity score matching provided approximate balance across the variables used to fit the propensity score model and additional variables not included as part of the propensity score model. However, some of the patient characteristics that were imbalanced (e.g., age) will require adjustment in the comparative effectiveness regression model to reduce residual confounding.

Our prospective cohort study compared patients with chronic musculoskeletal pain who were referred for acupuncture with those who were not referred for acupuncture. Some characteristics appeared imbalanced in the prospective cohort study based on their standardized differences, which were as large as -0.283 (e.g., age). In contrast, the overall balance was adequate based on the post-matching c-statistic (0.503), which exhibited little room for improvement relative to a score of 0.5 that would be expected with randomly-generated treatment assignments [12]. Although simulations have demonstrated that the post-matching c-statistic offers a valid assessment of covariate balance in retrospective cohort studies for which the propensity score is both developed and applied through matching in the same population, [13] we know little about its interpretation when the propensity score is transported to a distinct population with a different patient case-mix. Consequently, we emphasize the standardized differences in assessing the magnitude of imbalance.

This study adds important data to the growing body of literature describing interventions that used propensity scores [14, 15]. A recent systematic review of studies that applied propensity scores to control confounding reported that a minority (17%) of published studies evaluated clinical interventions other than surgery or medications [15]. We are aware of only two studies of acupuncture that have applied propensity scores techniques, and both studies applied propensity scores in retrospective studies (i.e., matching on characteristics and evaluating outcomes previously documented in the clinical record) [16, 17]. We think that our current study represents a unique application of propensity scoring.

Had it been feasible to compare the effectiveness of acupuncture (versus no acupuncture) based on an outcome that did not require collecting patient-reported outcomes (PROs), we could have conducted the cohort study on a broader population of treated and untreated patients without the need for ongoing matching, and then conducted traditional propensity score adjustment after the fact. That’s the typical scenario in retrospective cohort studies of comparative effectiveness or safety of treatments for pain: the outcome is collected during routine practice and documented at no expense to the investigators [18, 19].

Our prospective study required us to collect PROs before acupuncture treatment and repeatedly during the six-month follow-up. PROs require patients’ informed consent and are expensive to collect. As a result, we limited collection to those patients who met the eligibility criteria (which depended in part on patients’ PRO values preceding acupuncture treatment). While we enrolled all patients referred for acupuncture, we limited data collection of non-referred patients to those who were matched on decile of the propensity score. Using this design meant that we had to calculate each patient’s propensity score before enrollment in the prospective cohort, which was only possible using a propensity score developed using data from an historical cohort of similar patients; we hoped the historical propensity score would work well in our prospective cohort. The temporal validation of the propensity score and the approximate balance in the prospective cohort, as reported in the Results, support the effectiveness of this approach.

The matching worked despite several threats to its validity. First, the propensity score was developed to predict referral *and initiation* of acupuncture (i.e., adherence with at least one session). To serve the needs the prospective study, we had to enroll patients solely on the basis of *referral* for acupuncture (i.e., regardless of follow-up provision of acupuncture) in order to collect the needed baseline (pre-treatment) measurements. Because we recruited patients for a prospective study that required their consent to participate, our prospective cohort was subject to a potential selection bias that did not exist for the historical cohort used to develop the propensity score. Finally, we selected the first two controls patients who met criteria and agreed to participate to ensure that we could recruit an adequate number of non-referred, matched controls in a timely manner. As such, the non-referred patients who replied more quickly may differ in subtle ways from the larger pool of potential controls. This could introduce selection bias between those referred and not referred for acupuncture. While a combination of these factors could limit the effectiveness of the propensity score matching, our final referred and non-referred cohorts were approximately balanced.

Using propensity scores in prospective cohort studies is statistically and logistically feasible for health plans with electronic health records and integrated data. Their use, however, requires significant resources, including substantial effort during the period of recruitment, including ongoing analytic support. For example, information from a number of different data sources (EHR, online survey response databases, interviewer tracking system) had to be brought together in real time to identify potential comparison participants and enroll them in the study, which required a complex set of data transfers. The identification process could fail at various points. Accordingly, time devoted to building and maintaining this system, along with computational needs, were substantially greater than anticipated. Further, when system glitches occurred (sometimes due to unanticipated health service coding changes within the health care systems), recruitment processes needed to be suspended until the problem was adjudicated and systems were realigned. These challenges limited overall study recruitment.

While using this method was labor-intensive, the application of this new use of propensity score methodology may become increasingly efficient with more experience [20]. For example, we realized that we could build a somewhat simpler tracking system that would have increased the efficiency of the process. While such efficiencies are possible, the general feasibility of the approach appears limited to settings with electronic health records that will allow for the development of propensity scores (using historical data) and automated calculation of predicted treatment probabilities for recruitment and matching on a daily basis in prospective patients.

## Conclusion

Whenever investigators need to collect data prospectively in a cohort study evaluating comparative effectiveness, this approach may save time and money as well as reduce participant burden by collecting data only from patients who will contribute to the analysis. Prospective data collection is often required for behavioral health interventions, which depend on patient-reported covariates and outcomes, such as depression or pain. Prospective data collection is also required for many clinical studies, such as detailed clinical evaluations (e.g., the New York Heart Association class for heart failure), expensive laboratory tests, and diagnostic imaging. In these instances, prospective data collection may improve control of confounding as well as the assessment of treatment effect heterogeneity [21]. Our study demonstrated the feasibility of matching on a propensity score in a prospective cohort study before the start of treatment to improve the efficiency of data collection while approximately balancing the cohorts on a larger number of patient characteristics (to reduce confounding).

## Abbreviations

CMP: Chronic musculoskeletal pain
EHR: Electronic health record
HIPAA: Health Insurance Portability and Accountability Act
IRB: Institutional review board
KPNW: Kaiser Permanente Northwest
PRO: Patient-reported outcomes
